# Clinical Verifications

**Published:** 1891-05

**Authors:** George F. Dunham

**Affiliations:** Wenona, Ill.


### CLINICAL VERIFICATIONS.

GEORGE F. DUNHAM, WENONA, ILL.

NAT-MUR. IN INTERMITTENT FEVER.—Master Bob D. and sister Emma, aged respectively ten and fourteen years, were born and raised in a malarial district of Arkansas. Have been subject to attacks of ague for years. They had both taken large quantities of Quinine. Chill every other day about ten to eleven o'clock. Vomiting was very severe; in fact, the boy vomited blood a number of times. The first prescription was Sac-lac. until symptoms developed, which plainly called for Nat-mur. I gave the 15 dil. on pellets. A single prescription cured both cases.

BARYTA-CARB. IN TONSILLITIS.—Miss F. R., aged twenty-four, has had attacks of tonsillitis for past ten years. She was very susceptible to cold, the slightest exposure causing an attack. Tonsils enlarged and indurated. Has been treated by a homœopathic physician for past seven years, each attack resulting in suppuration. I told her I believed she could be cured. Gave Baryta-carb.$^{30x}$ trit., a dose once a week. She has had but one slight attack in three years, which was controlled promptly with Baryta-carb.$^{3x}$ without suppuration.

I have found Baryta-c. very successful in such cases, and failures very rare.

CHAMOMILLA IN COLIC.—Mr. J. B., aged twenty-six, was troubled for years with attacks of pain in the abdomen. During such attacks he was very cross, and felt as if he could not stand the pain. Warm applications would bring some relief, but he had to finally resort to Morphine. Each attack grew worse and came at shorter intervals. Considering the fact of his being a brother, I advised him to try *pure* Homœopathy. The symptoms were well marked. Chamomilla$^{3x}$, was given in doses at short intervals. Cure was prompt and permanent. It was his first trial of Homœopathy, and his last attack of colic, now ten years past.

SULPHUR$^{30x}$ IN CHRONIC DIARRHŒA.—Master F. E., aged twelve years, has been troubled for five years with an early morning diarrhœa, obliging him to arise at five o'clock each morning in great haste. Various remedies had been tried without benefit. Sulphur$^{30x}$ cured promptly. He has remained well for past three years.

NUX-V.$^{30x}$ IN RENAL COLIC.—Mr. B. L., aged forty-five, suffered from an attack of renal colic. Excruciating pain right side with unsuccessful desire for stool. Nux gave prompt relief.

